# The Protein Arginine Methyltransferase PRMT-5 Regulates SER-2 Tyramine Receptor-Mediated Behaviors in *Caenorhabditis elegans*

**DOI:** 10.1534/g3.118.200360

**Published:** 2018-05-14

**Authors:** Alexander Bowitch, Kerry L. Michaels, Michael C. Yu, Denise M. Ferkey

**Affiliations:** Department of Biological Sciences, University at Buffalo, The State University of New York, Buffalo, NY 14260

**Keywords:** tyramine, SER-2, GPCR (G protein-coupled receptor), protein arginine methylation, PRMT

## Abstract

G protein-coupled receptors are 7-pass transmembrane receptors that couple to heterotrimeric G proteins to mediate cellular responses to a diverse array of stimuli. Understanding the mechanisms that regulate G protein-coupled receptors is crucial to manipulating their signaling for therapeutic benefit. One key regulatory mechanism that contributes to the functional diversity of many signaling proteins is post-translational modification. Whereas phosphorylation remains the best studied of such modifications, arginine methylation by protein arginine methyltransferases is emerging as a key regulator of protein function. We previously published the first functional evidence that arginine methylation of G protein-coupled receptors modulates their signaling. We report here a third receptor that is regulated by arginine methylation, the *Caenorhabditis elegans* SER-2 tyramine receptor. We show that arginines within a putative methylation motif in the third intracellular loop of SER-2 are methylated by PRMT5 *in vitro*. Our data also suggest that this modification enhances SER-2 signaling *in vivo* to modulate animal behavior. The identification of a third G protein-coupled receptor to be functionally regulated by arginine methylation suggests that this post-translational modification may be utilized to regulate signaling through a broad array of G protein-coupled receptors.

One way in which G protein-coupled receptor (GPCR) signaling can be regulated is through post-translational modification of receptors. Although a large-scale proteomics analysis performed in 2003 identified several GPCRs as substrates of an anti-methylarginine antibody (Boisvert *et al.* 2003), there was no direct evidence at that time that this receptor class is regulated by methylation. In 2015, we reported the first functional evidence that protein arginine methylation regulates GPCR signaling (Likhite *et al.* 2015*)*. Methylation of D2-like dopamine receptors (human D2 and *C. elegans* DOP-3) by protein arginine methyltransferase 5 (human PRMT5 and *C. elegans* PRMT-5, respectively) promoted signaling in both cell culture and *in vivo* (Likhite *et al.* 2015). The D2-like dopamine receptor family was identified as a possible substrate for PRMT5 in a bioinformatics analysis that examined GPCRs for predicted methylation motifs (RGG or RXR) in their intracellular domains. The human D2 receptor was found to have a putative methylation motif in its third intracellular loop that is conserved in the corresponding receptor sequences from other mammalian, vertebrate and invertebrate species, including the corresponding *C. elegans* D2-like dopamine receptor, DOP-3. The third intracellular loop of both the human D2 and *C. elegans* DOP-3 receptors was methylated by human PRMT5 *in vitro*, and changing the conserved arginines of the putative methylation motif to alanines diminished receptor methylation. Correspondingly, changing these arginines to disrupt the methylation motif also diminished signaling through both of these receptors (Likhite *et al.* 2015). Combined, the results of that study revealed that arginine methylation promotes signaling through the D2 receptor to dampen cAMP signaling in cultured human cells, and also promotes DOP-3 signaling to regulate *C. elegans* behavior.

PRMT5 is a type 2 protein arginine methyltransferase (PRMT) that transfers two methyl groups from S-adenosyl-L-methionine (SAM) to form symmetric dimethylarginines (SDMAs) (Branscombe *et al.* 2001). This modification can be added to arginines in glycine- and arginine-rich motifs, in proline-, glycine-, and methionine-rich motifs, and even in the absence of any recognizable motif (Bedford and Clarke 2009; Wang *et al.* 2013; Wang *et al.* 2014). PRMT5 has been shown to influence gene expression, snRNP biogenesis, the DNA damage response, and germ cell development (Meister *et al.* 2001; Fabbrizio *et al.* 2002; Ancelin *et al.* 2006; Tee *et al.* 2010; He *et al.* 2011; Huang *et al.* 2011). In their study of D2-like dopamine receptors, Likhite *et al.* (2015) added to the growing list of PRMT5 substrates and described the founding members of a new class of proteins – GPCRs – that are functionally regulated by arginine methylation.

The bioinformatics analysis performed by Likhite *et al.* (2015) identified 300 human GPCRs and 64 *C. elegans* GPCRs that contain an intracellular RGG or RXR putative methylation motif. Many of the identified *C. elegans* receptors are predicted or known to bind biogenic amines including dopamine, serotonin, octopamine and tyramine (Chase and Koelle 2007). In humans, tyramine has been considered a trace amine because it is found at low levels. However, a new family of GPCRs, the trace amine-associated receptors (TAARs), was discovered in 2001, suggesting that tyramine may act as a classical neurotransmitter in vertebrates (Borowsky *et al.* 2001). In addition, there is evidence that tyramine plays an important physiological role in humans and has been linked to human disorders such as hypertensive crisis and attention deficit hyperactivity disorder (ADHD) (Blackwell and Mabbitt 1965; Burchett and Hicks 2006; Berry 2007; D’Andrea *et al.* 2013). In *C. elegans*, tyramine also acts as a neurotransmitter and is considered the invertebrate counterpart of adrenaline (Roeder *et al.* 2003; Alkema *et al.* 2005; Roeder 2005). Once thought to act only as the precursor to octopamine, it is now clear that tyramine signaling modulates numerous *C. elegans* behaviors, ranging from the inhibition of egg laying to the formation and retrieval of imprinted memories (Rex *et al.* 2004; Alkema *et al.* 2005; Rex *et al.* 2005; Chase and Koelle 2007; Wragg *et al.* 2007; Pirri *et al.* 2009; Ringstad *et al.* 2009; Donnelly *et al.* 2013; Jin *et al.* 2016).

The *C. elegans* genome encodes three tyraminergic GPCRs, SER-2, TYRA-2 and TYRA-3, and one ligand-gated ion channel, LGC-55, that bind tyramine (Rex and Komuniecki 2002; Tsalik *et al.* 2003; Rex *et al.* 2004; Wragg *et al.* 2007; Pirri *et al.* 2009; Donnelly *et al.* 2013). The most extensively characterized of the GPCRs is the SER-2 receptor, which is expressed in a subset of sensory neurons, interneurons and motor neurons, as well as head muscles and pharyngeal cells (Altun-Gultekin *et al.* 2001; Rex and Komuniecki 2002; Tsalik *et al.* 2003; Rex *et al.* 2004; Alkema *et al.* 2005; Donnelly *et al.* 2013; Wilson *et al.* 2017). Among tyramine-regulated behaviors, a role for SER-2 has been shown in mediating tyramine (TA) -induced immobilization (Donnelly *et al.* 2013) and in antagonizing serotonin (5-HT) -stimulated pharyngeal pumping (Rex *et al.* 2004). In these studies, *ser-2 loss-of-function (lof)* animals were resistant to the paralytic effects of exogenous TA (Donnelly *et al.* 2013) and the addition of TA did not antagonize 5-HT-stimulated pumping in *ser-2(lof)* animals (Rex *et al.* 2004), respectively. In both cases, expression of a wild-type *ser-2* transgene rescued the behavioral phenotypes, demonstrating that they were specific to the loss of SER-2 receptor function.

While exploring their environments during forward locomotion, *C. elegans* display a foraging behavior in which they move their nose from side-to-side (Croll and Smith 1978). This foraging behavior is inhibited while animals reverse in response to light anterior mechanosensory stimulation, termed anterior touch (Chalfie *et al.* 1985; Alkema *et al.* 2005). Suppression of head movements while reversing in response to touch could help an animal escape from nematophagous fungi that can trap worms with constricting hyphal rings (Barron 1977). Extensive circuit-level analyses have revealed a critical role for TA and LGC-55 in suppressing foraging behavior in response to anterior touch; animals unable to synthesize TA and animals lacking LGC-55 do not suppress head oscillations during this backing response (Alkema *et al.* 2005; Pirri *et al.* 2009). Although not seen by Alkema *et al.* (2005), it has been reported that animals also suppress foraging while reversing in response to nose touch (Rex *et al.* 2004). The explanation for the difference is not clear, but could be the result of nuanced differences in the execution of the nose touch assay. Rex *et al.* (2004) found that *ser-2(lof)* animals continued to display foraging behavior while reversing following nose touch, unlike the wild-type animals in their study, and expression of a wild-type *ser-2* transgene rescued the behavioral phenotype (Rex *et al.* 2004). We also have observed that, in contrast to wild-type animals, *ser-2(lof)* animals do not cease foraging while backing in response to nose touch.

Following the bioinformatics analysis of Likhite *et al.* (2015), it was unknown if any of the other GPCRs identified to contain an intracellular RGG or RXR putative methylation motif are functionally regulated by arginine methylation, similar to the D2-like dopamine receptors. Herein, we report that the *C. elegans* SER-2 tyramine receptor is also regulated by methylation. We show that human PRMT5 methylates a portion of the third intracellular loop of SER-2 *in vitro*, and that the conserved arginines within the predicted methylation motif are required for methylation by PRMT5. Using *C. elegans* behavior as a readout for nervous system function, we show that PRMT-5 also promotes tyraminergic signaling through the *C. elegans* SER-2 receptor *in vivo*, and that changing the predicted arginine methylation target sites in SER-2 diminished its ability to regulate *C. elegans* behavior. Together, our data reveal a third receptor that appears to be functionally regulated by protein arginine methylation. This work suggests that arginine methylation may be a widespread post-translational modification used to regulate the activity of GPCRs.

## Materials And Methods

### C. elegans Culture

Strains were maintained at 20° under standard conditions on nematode growth media (NGM) agar plates seeded with OP50 *E. coli* bacteria (Brenner 1974).

### Strains

Strains used in this study include: N2 Bristol wild-type, OH313 *ser-2(pk1357)*, FG129 *prmt-5(gk357)*, FG807 *ser-2(pk1357)*;*prmt-5(gk357)*, FG808 *ser-2(pk1357);udEx460[ser-2p::ser-2,elt-2::GFP]*, FG809 *ser-2(pk1357);udEx461[ser-2p::ser-2,elt-2::GFP]*, FG810 *ser-2(pk1357);udEx462[ser-2p::ser-2,elt-2::GFP]*, FG811 *ser-2(pk1357);udEx463[ser-2p::ser-2(R245A/R247A),elt-2::GFP]*, FG812 *ser-2(pk1357);udEx464[ser-2p::ser-2(R245A/R247A),elt-2::GFP]*, FG813 *ser-2(pk1357);udEx465[ser-2p::ser-2(R245A/R247A),elt-2::GFP]*, FG814 *prmt-5(gk357);udEx466[ser-2p::prmt-5,elt-2::GFP]*, FG815 *prmt-5(gk357);udEx467[ser-2p::prmt-5,elt-2::GFP]* and FG816 *prmt-5(gk357);udEx468[ser-2p::prmt-5,elt-2::GFP]*.

### Transgenic Strains

Germline transformations were performed as previously described (Mello *et al.* 1991). For *prmt-5* and *ser-2* rescue experiments, pJM67 *elt-2*::*gfp* plasmid (25 ng/μl) (Fukushige *et al.* 1998) was used as the co-injection marker, along with either the *prmt-5* or *ser-2* rescuing plasmid (50 ng/μl).

### Plasmid Construction

#### pFG2:

The ∼5.3 kb *glr-1* promoter was cut out of C06E1xP, first by digesting with *Sal*I and blunting with Klenow, followed by digestion with *Pst*I. This fragment was gel purified and ligated into the *Pst*I/*Sma*I sites of pPD49.26 (Fire Lab *C. elegans* Vector Kit, Addgene).

#### pFG102:

The *ser-2* cDNA (isoform e) was PCR amplified from *ser-2* in pFLAG (gift from Rick Komunicki) (Rex *et al.* 2004) with primers designed to incorporate a 5′ *Kpn*I site and a 3′ *Sac*I site, and subcloned into these sites of pFG2.

#### pFG288:

The ∼2.2 kb *ser-2* promoter (Donnelly *et al.* 2013) was PCR amplified from N2 genomic DNA, incorporating a 5′ *Bam*HI and a 3′ *Hin*dIII site, and subcloned into these sites of pPD49.26 (Fire Lab *C. elegans* Vector Kit, Addgene).

#### pFG289:

The cDNA encoding *ser-2* (isoform e) was isolated from pFG102 with *Kpn*I/*Sac*I, and subcloned into these sites of pPD49.26 (Fire Lab *C. elegans* Vector Kit, Addgene).

#### pFG290 ser-2p::ser-2:

The ∼2.2 kb *ser-2* promoter (Donnelly *et al.* 2013) was PCR amplified from N2 genomic DNA, incorporating a 5′ *Bam*HI and a 3′ *Hin*dIII site, and subcloned into these sites of pFG289.

#### pFG291 ser-2p::ser-2(R245A/R247A):

Site-directed mutagenesis (QuikChange, Stratagene) was used to incorporate the R245A and R247A substitutions into the *ser-2p*::*ser-2* plasmid pFG290.

#### pFG292 ser-2p::prmt-5:

The cDNA encoding *prmt-5* was isolated from pFG66 (Likhite *et al.* 2015) with *Nhe*I/*Kpn*I, and inserted into these sites of pFG288.

#### pFG293 GST-S-SER-2 3ICL-S 30 aa:

A portion of the cDNA encoding the third intracellular loop (ICL) of SER-2 (amino acid residues 240 to 269 of isoform e) was amplified by PCR from *ser-2p*::*ser-2* (pFG290), incorporating sequence encoding an N- and C-terminal S-tag (amino acids KETAAAKFERQHMDS) as well as a 5′ *Bam*HI and a 3′ *Xma*I site. This DNA was then inserted into the corresponding sites of pGEX-5X-3.

#### pFG294 GST-S-SER-2(R245A/R247A) 3ICL-S 30 aa:

A portion of the cDNA encoding the third intracellular loop (ICL) of SER-2(R245A/R247A) (amino acid residues 240 to 269 of isoform e) was amplified by PCR from *ser-2p*::*ser-2(R245A/R247A)* (pFG291), incorporating sequence encoding an N- and C-terminal S-tag (amino acids KETAAAKFERQHMDS) as well as a 5′ *Bam*HI and a 3′ *Xma*I site. This DNA was then inserted into the corresponding sites of pGEX-5X-3.

All constructs were verified by sequencing where appropriate.

### Behavioral Assays

All behavioral assays were performed on at least three separate days, in parallel with controls. Assays were performed at room temperature using young adult animals aged 24 hr post the L4 larval stage. For all behavioral experiments the combined data of ≥ 3 transgenic lines is shown, and the number of transgenic animals assayed in each experiment is indicated within the figure legends. In all cases n ≥ 24 for non-transgenic animals. The Student’s two-tailed t-Test and one-way Anova with Tukey’s Honestly Significant Difference (HSD) were used for statistical analyses.

To quantify resistance to tyramine-induced immobilization, young adult animals were transferred to agar plates supplemented with 12 mM tyramine. Approximately 8 animals were transferred to assay plates and scored for locomotion every minute for a 10 min period. Animals were scored as immobilized if there was no sustained forward or backward locomotion in a 5 sec interval (Donnelly *et al.* 2013). Tyramine plates were prepared by autoclaving 1.7% agar in water, cooling to ∼55° and adding glacial acetic acid to a concentration of 2 mM and tyramine-hydrochloride (Sigma-Aldrich) to a concentration of 12 mM.

Pharyngeal pumping was measured by washing young adult animals aged 24 hr post the L4 larval stage off of a seeded plate with M9 buffer. Animals were washed twice in M9 buffer (Wood 1988) and then incubated in ligand for 20 min. Animals were then spun down and transferred onto agar pads and the number of pumps per 20 sec was counted. A pump was defined as the movement of the pharyngeal grinder. The ligands tyramine-hydrochloride (Sigma-Aldrich) and serotonin creatinine sulfate monohydrate (Sigma-Aldrich) were prepared at the indicated concentrations in M9 buffer.

The nose touch assay was performed essentially as described (Kaplan and Horvitz 1993; Hart *et al.* 1995). Briefly, young adult animals were transferred to agar plates spread with 100 μl OP50 and allowed to recover for 5 min. An arm hair was placed in the path of a forward-moving animal to allow a “nose-on” collision. The presence or absence of foraging (classified as the continuous movement of the head in an exploratory fashion) while reversing was recorded. Five trials per animal and ≥30 animals per genotype were scored as follows: no foraging (no head movement while reversing) and foraging (head movement while reversing). Occasionally, animals did not reverse upon nose touch and were scored as no response.

### Protein Purification

Overnight cultures of *E. coli* expressing either GST, GST-SER-2_240-269_ [GST-S-SER-2 3ICL-S (30 amino acids)] or GST-SER-2_240-269_(R245A/R247A) [GST-S-SER-2(R245A/R247A) 3ICL-S (30 amino acids)] were pelleted, resuspended in lysis buffer (1x PBS/1 M NaCl, 1 mM PMSF, 1 mM DTT, 1 mg/ml lysozyme) and subjected to two rounds of French Press lysis. Lysates was centrifuged at 30,000 × g for 30 min at 4°. Supernatants were incubated with Glutathione Sepharose High Performance beads (Amersham Biosciences) that had been equilibrated with wash buffer (1x PBS/1 M NaCl, 0.02% v/v Triton X-100, 1 mM DTT). Unbound sample was allowed to flow through and the resin was washed 3x with 10 mL of wash buffer. GST-tagged proteins were eluted with elution buffer (50 mM Tris pH 8, 200 mM NaCl, 0.01% v/v Triton X-100, 1 mM DTT, 15 mM glutathione). Fractions containing the protein eluate were dialyzed in dialysis buffer (1x PBS, 15% glycerol) for storage at -80°.

### In vitro Methylation

The *in vitro* methylation assay was performed essentially as described (Likhite *et al.* 2015*)* in a total volume of 20 μl with 6 μg of substrate (50 ng of SmB’ (Goulet *et al.* 2007)), 210 ng of recombinant human PRMT5 complex (Active Motif), and 5.5 μCi of S-[methyl-^3^H]adenosyl-L-methionine (55 to 85 Ci/mmol; PerkinElmer) in 1x methylation buffer [150 mM NaCl, 50 mM Tris-HCl (pH 8), 1 mM EDTA]. Reactions were incubated at 37° for 4 hr, resolved by SDS-polyacrylamide gel electrophoresis, and then transferred to polyvinylidene difluoride (PVDF) membranes. The membranes were sprayed with 6% PPO enhance reagent (2,5-Diphenyloxazole, in isopropanol) three times at 10 min intervals before being exposed to Kodak BioMax MS film with a BioMax Transcreen LE Intensifying Screen at -80° for two weeks and subsequently developed. Band intensities were quantified with Bio-Rad ImageLab software and were normalized according to gel loading.

### Western Blotting

Following film exposure for quantification of methylation signal, the PVDF membrane was washed two times with 100% methanol and two times in PBS-T to remove the PPO. The washed membrane was then blocked with blocking solution (5% milk in PBS-T) for one hour at room temperature. Polyclonal anti-GST primary antibody (Abcam, ab19256) was used at a 1:20,000 dilution. The secondary antibody (horseradish peroxidase-conjugated light-chain specific mouse anti-rabbit IgG, Bio-Rad) was used at a 1:10,000 dilution. The chemiluminescent reaction was performed using Amersham ECL Prime Western Blotting Detecting Reagent (GE Healthcare).

### Bioinformatics Analysis

TMpred (Prediction of Transmembrane Regions and Orientation) (Hofmann and Stoffel 1993) and TMHMM (Predication of Transmembrane Helices in Proteins, v2.0) (Krogh *et al.* 2001) were used (with the default model parameters) to predict the locations of transmembrane domains (TMDs).

### Data and Reagent Availability

Strains and plasmids are available upon request. The authors affirm that all data necessary for confirming the conclusions of this article are represented fully within the article and its figures. Figure S1 shows the sequence alignments of the predicted arginine methylation motifs from related receptors, as included in the Discussion. Supplemental material available at Figshare: https://doi.org/10.25387/g3.6193037.

## Results

### PRMT5 methylates the C. elegans SER-2 receptor

**Figure 1:**
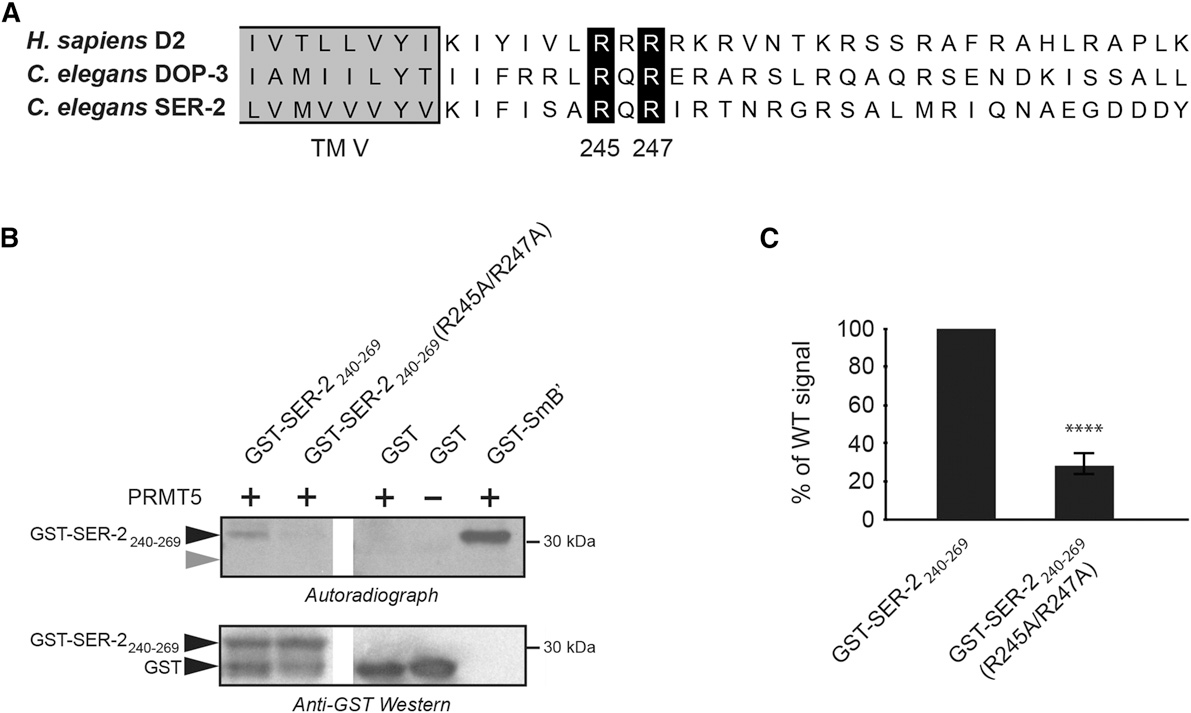
Human PRMT5 methylates the *C. elegans* SER-2 receptor *in vitro*. (A) Alignment showing conservation of the predicted arginine methylation motifs of the human (D2) and *C. elegans* (DOP-3) D2-like dopamine receptors, along with the *C. elegans* SER-2 tyramine receptor. Resides Arg^245^ and Arg^247^ of SER-2 are indicated and lie within a conserved RXR motif, in which X can be any amino acid residue. The entire third intracellular loop of SER-2 is comprised of 133 amino acids (representing residues 239-371); only residues 239-269 of the third intracellular loop are shown. The gray shading indicates the end of transmembrane domain five (TM V) of the receptors. (B) Representative blot for the *in vitro* methylation assay. A wild-type and mutant recombinant fragment of the third intracellular loop of the *C. elegans* SER-2 receptor [amino acid residues 240-269, flanked both amino- and carboxy- terminally with an S-tag to increase solubility, and fused to glutathione *S*-transferase (GST)] were used in an *in vitro* methylation assay with active recombinant human PRMT5. There are no arginines within the S-tag. A GST-tagged portion of SmB’ protein, a robust PRMT5 substrate (Goulet *et al.* 2007), served as the positive control and GST was used as the negative control. The autoradiograph shows that the wild-type GST-SER-2_240-269_ fragment was methylated by PRMT5, while methylation of GST-SER-2_240-269_(R245A/R247A) was significantly diminished (*P* ≤ 0.0001). Gray arrowhead indicates the molecular weight position of GST in the autoradiograph, which was not methylated. Anti-GST Western blotting of the polyvinylidene difluoride (PVDF) membrane was used to normalize values for equivalent substrate loading. Figure panels were made from a single exposure of the membrane; lanes unrelated to this study were cut from the image. GST-SmB’ does not appear on the Western because much less was used as substrate and loaded relative to the other lanes. Molecular mass markers (kDa) are indicated on the right. (C) Quantification of the degree of methylation of the receptor fragments was based on densitometric analysis of the autoradiographs. The degree of GST-SER-2_240-269_(R245A/R247A) methylation was 30% of that of the wild-type (WT) fragment. Error bar represents the standard error of the mean (SEM) from three independent experiments. **** *P* ≤ 0.0001.

We previously reported the PRMT5-dependent methylation of two D2-like dopamine GPCRs, the human D2 and *C. elegans* DOP-3 receptors (Likhite *et al.* 2015). These receptors contain an arginine methylation motif in the third intracellular loop that is highly conserved across species (Likhite *et al.* 2015). We wished to investigate the possible methylation of another GPCR, the *C. elegans* SER-2 tyramine receptor. SER-2 has a predicted methylation motif in the third intracellular loop with identical placement to those seen in the human D2 and *C. elegans* DOP-3 receptors (Figure 1A). To test the ability of the methyltransferase PRMT5 to methylate the SER-2 receptor, we performed an *in vitro* methylation assay. A recombinant fragment of the third intracellular loop of the SER-2 receptor [amino acid residues 240-269, flanked both amino- and carboxy- terminally with an S-tag to increase solubility, and fused to glutathione *S*-transferase (GST)] was methylated by PRMT5 (Figure 1B). To determine whether the arginines of the predicted methylation motif (Arg^245^ and Arg^247^) were necessary for SER-2 methylation, we generated a recombinant fragment in which these arginines were changed to alanines (R245A/R247A). SER-2 receptor methylation was markedly diminished when the two conserved arginine residues were replaced with alanines (Figure 1B). Quantification of the bands from three independent experiments showed that less than 30% of the wild-type SER-2_240-269_ signal was present when SER-2_240-269_(R245A/R247A) was used as substrate (Figure 1C). These data establish the third intracellular loop of the SER-2 receptor as a substrate for PRMT5 *in vitro* and suggest that Arg^245^ and Arg^247^ are key sites of methylation within this region.

### C. elegans PRMT-5 contributes to the regulation of locomotion by exogenous tyramine

Having identified the SER-2 receptor as a substrate for PRMT5-mediated methylation *in vitro*, we wished to determine the extent to which arginine methylation affects SER-2 signaling *in vivo*. To do this, we examined *C. elegans* behaviors that are modulated by tyramine signaling through the SER-2 receptor, in animals lacking the protein arginine methyltransferase PRMT-5.

**Figure 2:**
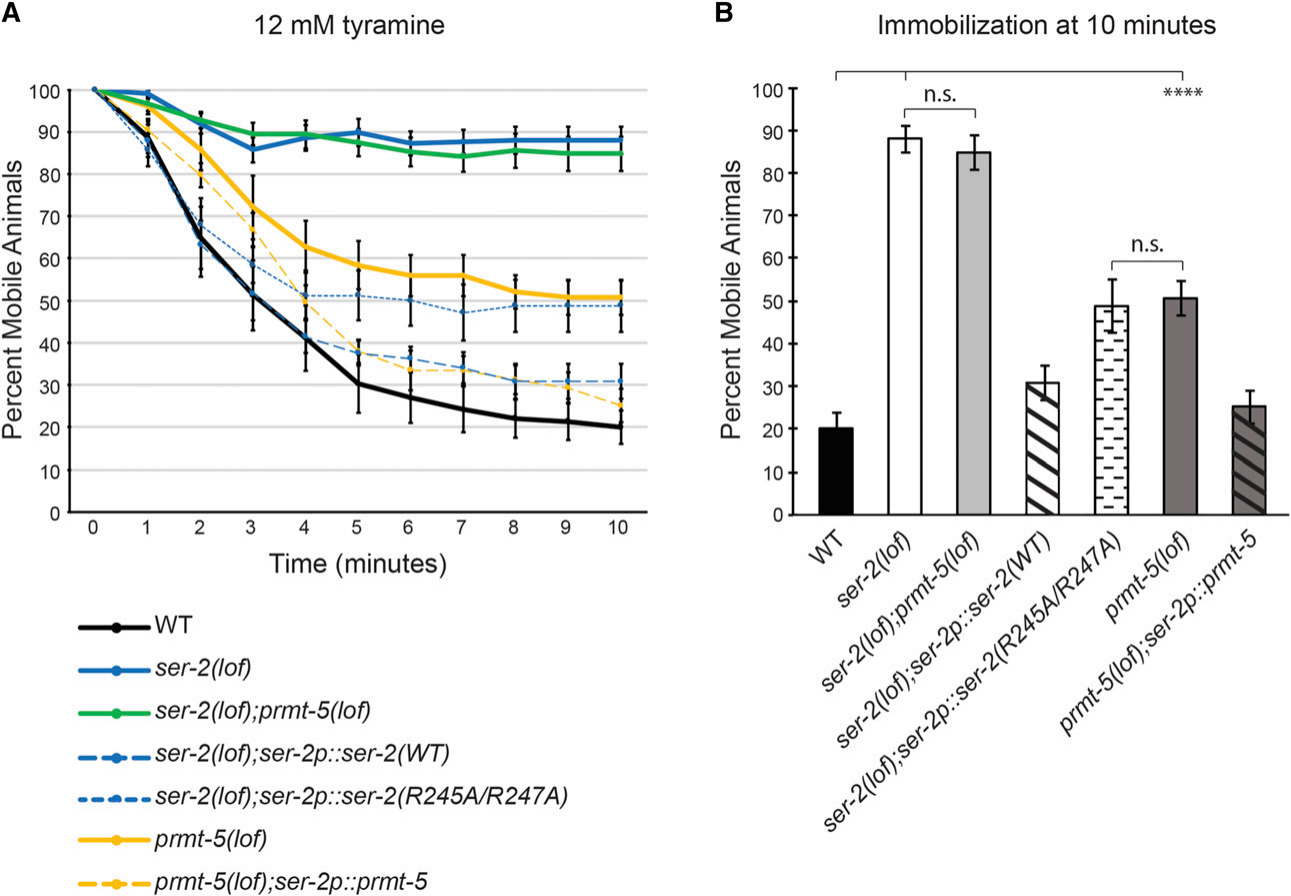
*C. elegans* lacking PRMT-5 are less susceptible to tyramine-induced immobilization. (A) *prmt-5* loss-of-function animals display an intermediate immobilization phenotype when compared to wild-type and *ser-2(lof)* animals (*P* ≤ 0.001 when comparing *prmt-5(lof)* animals to either *ser-2(lof)* or wild-type animals across time). Restoring WT SER-2 function (*ser-2p*::*ser-2)* fully rescued tyramine-induced immobilization (*P* > 0.2 when compared to wild-type animals across time). *ser-2(lof)* animals expressing SER-2(R245A/R247A) displayed a partial resistance to tyramine-induced immobilization, similar to *prmt-5(lof)* animals (*P* > 0.8 across time). The percentage of mobile animals on 12 mM tyramine plates at each time-point is shown. (B) The bar graph shows the percentage of animals that became immobilized on 12 mM tyramine plates at the 10 min endpoint displayed in panel A. Alleles used: *prmt-5(gk357)* and *ser-2(pk1357)*. WT = the N2 wild-type strain. For rescue experiments, the combined data of three independent transgenic lines and n ≥ 78 transgenic animals are shown. Error bars represent the standard error of the mean (SEM). **** *P* ≤ 0.0001. n.s. = not significant.

Tyramine (TA) modulates *C. elegans* locomotor behavior by activating SER-2 in the GABAergic motor neurons (Donnelly *et al.* 2013). Wild-type animals become immobilized on plates containing exogenous tyramine, while *ser-2(lof)* animals are resistant to this paralysis (Donnelly *et al.* 2013). To determine if protein arginine methylation contributes to TA-induced immobilization through SER-2 signaling *in vivo*, we tested the effect of exogenous TA on animals lacking PRMT-5. *prmt-5(lof)* animals displayed an intermediate level of immobilization when compared to wild-type and *ser-2(lof)* animals (Figure 2). Animals lacking both the SER-2 receptor and PRMT-5 [*ser-2(lof);prmt-5(lof)* double mutants] displayed immobilization levels similar to those of the s*er-2(lof)* single mutants, suggesting that these two proteins function in the same pathway. The partial tyramine resistance observed in *prmt-5(lof)* animals is consistent with PRMT-5 playing a role in promoting SER-2-mediated tyramine signaling.

To determine whether PRMT-5 regulates tyramine-modulated paralysis by acting in the same cells as SER-2, we used the *ser-2* promoter (*ser-2p*, 2.2 kb upstream of the first translational start site (Donnelly *et al.* 2013)) to drive *prmt-5* cDNA expression and restore PRMT-5 function in *ser-2*-expressing cells. *prmt-5(lof)* animals expressing the *ser-2p*::*prmt-5* transgene displayed an immobilization phenotype similar to that of wild-type animals (Figure 2). Using the *ser-2* promoter to restore SER-2 (isoform e) receptor expression [*ser-2p*::*ser-2(WT)*] in *ser-2(lof)* animals also fully rescued TA-induced immobilization (Figure 2). To determine if the arginines of the predicted PRMT-5 methylation motif (Arg^245^ and Arg^247^) contributed to SER-2 function *in vivo*, we generated a SER-2(R245A/R247A) mutant receptor using site-directed mutagenesis. *ser-2(lof)* animals expressing the *ser-2p*::*ser-2(R245A/R247A)* transgene phenocopied *prmt-5(lof)* animals (Figure 2). Taken together, these results suggest that Arg^245^ and Arg^247^ in the predicted PRMT-5 methylation motif of the third intracellular loop of the SER-2 receptor contribute to its signaling potential *in vivo*. Our data are consistent with methylation of these arginines by PRMT-5 playing a role in promoting SER-2 signaling.

### C. elegans PRMT-5 promotes tyramine-mediated inhibition of serotonin-stimulated pharyngeal pumping

Pharyngeal pumping is a cycle of contraction and relaxation of the pharyngeal muscle that transports bacteria from the pharynx of the worm into its intestine. Exogenous serotonin (5-HT) can stimulate pharyngeal pumping by mimicking the presence of food, while the addition of exogenous TA antagonizes 5-HT-stimulated pumping (Horvitz *et al.* 1982). However, consistent with SER-2 expression in pharyngeal muscles (Tsalik *et al.* 2003), TA does not inhibit 5-HT-stimulated pumping in *ser-2(lof)* animals (Rex *et al.* 2004).

**Figure 3:**
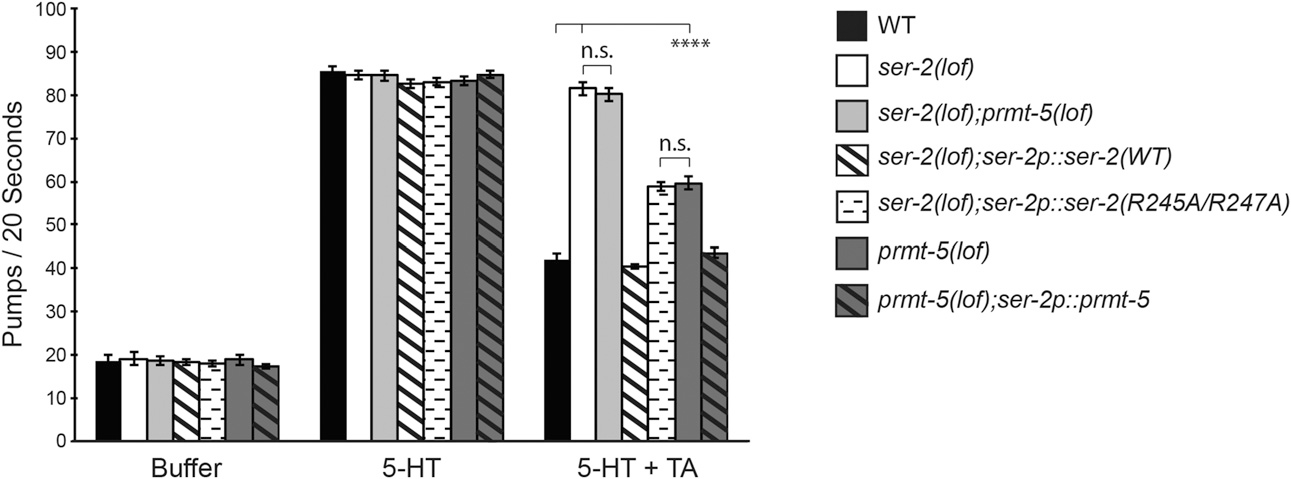
*C. elegans* PRMT-5 promotes TA inhibition of 5-HT-stimulated pharyngeal pumping. Animals were incubated in M9 buffer, 5-HT (10 mM), or TA (2 mM) + 5-HT (10 mM). The number of pumps per 20 sec was counted. In the presence of 5-HT + TA, *prmt-5(lof)* animals displayed an intermediate pharyngeal pumping phenotype when compared to wild-type and *ser-2(lof)* animals (*P* ≤ 0.0001 when comparing *prmt-5(lof)* animals to either *ser-2(lof)* or wild-type animals). *prmt-5(lof)* animals expressing *ser-2p*::*prmt-5* showed a pharyngeal pumping rate similar to wild-type animals (*P* > 0.5) in the presence of 5-HT and TA. Restoring WT SER-2 function [*ser-2p*::*ser-2(WT)*] fully rescued TA-mediated inhibition of 5-HT stimulation (*P* > 0.07). *ser-2(lof)* animals expressing SER-2(R245A/R247A) displayed a partial TA-mediated inhibition, similar to *prmt-5(lof)* animals (*P* > 0.6). The pumps per 20 sec is shown. Alleles used: *prmt-5(gk357)* and *ser-2(pk1357)*. WT = the N2 wild-type strain. For rescue experiments, the combined data of three independent transgenic lines and n ≥ 42 transgenic animals are shown. Error bars represent the standard error of the mean (SEM). **** *P* ≤ 0.0001. n.s. = not significant.

To determine if protein arginine methylation promotes TA-mediated inhibition of 5-HT-stimulated pharyngeal pumping, we first tested the effect of exogenous TA on animals lacking PRMT-5 function. Similar to the TA-induced immobilization experiments (Figure 2), *prmt-5(lof)* animals displayed an intermediate pharyngeal pumping phenotype when compared to wild-type and *ser-2(lof)* animals (Figure 3). *prmt-5(lof)* animals expressing the *ser-2p*::*prmt-5* transgene to restore PRMT-5 function in *ser-2*-expressing cells showed a pharyngeal pumping rate similar to wild-type animals in the presence of 5-HT and TA, consistent with PRMT-5 regulating pharyngeal pumping by acting in the same cells as SER-2. To assess the contribution of Arg^245^ and Arg^247^ to SER-2 function, we compared TA inhibition of 5-HT-stimulated pharyngeal pumping in *ser-2(lof)* animals expressing the *ser-2p*::*ser-2(WT)* *vs.* *ser-2p*::*ser-2(R245A/R247A)* transgene. While *ser-2(lof)* animals expressing wild-type SER-2 displayed a pumping rate similar to wild-type animals, *ser-2(lof)* animals expressing SER-2(R245A/R247A) were again similar to animals lacking PRMT-5 (Figure 3). Combined, these results suggest that PRMT-5 promotes SER-2 signaling in pharyngeal cells, and that the two arginines in the predicted methylation motif are important for SER-2 receptor signaling *in vivo*.

### Loss of PRMT-5 function leads to continued foraging behavior in response to nose touch

**Figure 4:**
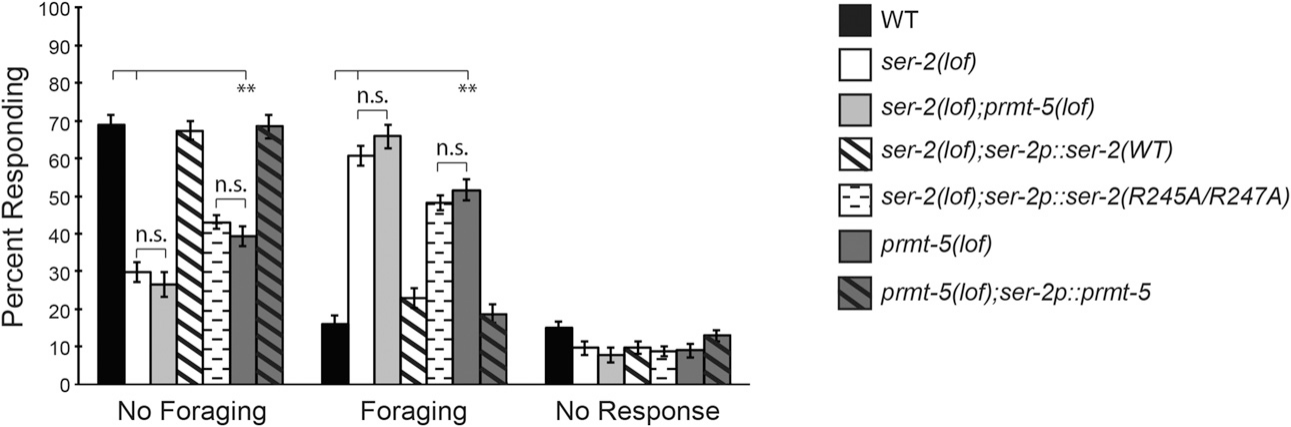
*C. elegans* PRMT-5 suppresses foraging in response to nose touch. The presence or absence of foraging behavior following nose touch was scored during the reversal response. *prmt-5(lof)* animals displayed an intermediate foraging phenotype when compared to wild-type and *ser-2(lof)* animals (*P* ≤ 0.01 when comparing *prmt-5(lof)* animals to either *ser-2(lof)* or wild-type animals). *prmt-5(lof)* animals expressing *ser-2p*::*prmt-5* showed a foraging rate similar to wild-type animals (*P* > 0.8). Restoring wild-type SER-2 function [*ser-2p*::*ser-2(WT)*] in *ser-2(lof)* animals fully rescued the suppression of foraging (*P* > 0.6 when compared to wild-type animals). *ser-2(lof)* animals expressing SER-2(R245A/R247A) displayed an intermediate degree of foraging, similar to *prmt-5(lof)* animals (*P* > 0.2). No Foraging = inhibition of head movement while reversing; Foraging = continuous head movement while reversing; No Response = no reversal upon nose touch. Alleles used: *prmt-5(gk357)* and *ser-2(pk1357)*. WT = the N2 wild-type strain. For rescue experiments, the combined data of three independent transgenic lines and n ≥ 45 transgenic animals (5 trials per animal) are shown. Error bars represent the standard error of the mean (SEM). ** *P* ≤ 0.01. n.s. = not significant.

Consistent with SER-2 expression in both the neurons and muscles of the head that affect head movement (Tsalik *et al.* 2003; Rex *et al.* 2004; Donnelly *et al.* 2013; Wilson *et al.* 2017), *ser-2(lof)* animals fail to suppress foraging behavior while reversing in response to nose touch (Rex *et al.* 2004). To determine if protein arginine methylation regulates *C. elegans* foraging behavior, we first assessed the extent to which *prmt-5(lof)* animals suppress foraging in response to nose touch. While only 16% of wild-type animals continued foraging while reversing, 51% of *prmt-5(lof)* animals continued foraging as they reversed following nose touch (Figure 4). By comparison, 60% of *ser-2(lof)* animals did not suppress foraging while reversing. Restoration of PRMT-5 function in *ser-2*-expressing cells of *prmt-5(lof)* animals (by expressing the *ser-2p*::*prmt-5* transgene) suppressed foraging to the extent seen in wild-type animals. Furthermore, while expression of wild-type SER-2 in *ser-2(lof)* animals suppressed foraging to wild-type levels, *ser-2(lof)* animals expressing SER-2(R245A/R247A) did not fully suppress foraging, similar to *prmt-5(lof)* animals. Taken together, these data are consistent with a role for arginine methylation promoting SER-2 signaling to dampen foraging behavior in response to nose touch.

## Discussion

Our lab previously reported the first functional evidence that signaling through GPCRs, specifically D2-like dopamine receptors (human D2 and *C. elegans* DOP-3), is regulated by protein arginine methylation (Likhite *et al.* 2015). Herein, we provide evidence that a third GPCR, the *C. elegans* SER-2 tyramine receptor, is also functionally regulated by protein arginine methylation. We show that human PRMT5 methylates the third intracellular loop of the SER-2 receptor *in vitro*, and that mutating the conserved arginines (Arg245/Arg247) of the putative methylation motif reduced this methylation (Figure 1). *C. elegans* PRMT-5 also promotes SER-2 regulated behaviors *in vivo*, and changing the conserved arginines of SER-2 reduced its ability to regulate these behaviors (Figures 2-4). This work, combined with our previous study (Likhite *et al.* 2015), suggests that protein arginine methylation may serve as an important regulatory means to modulate the activity of a diversity of GPCRs.

Natural predators of wild *C. elegans* include nematophagous fungi (Barron 1977) armed with constricting hyphal rings that can capture worms (Thorn andBarron 1984). Mechanosensory stimulation of the hyphal rings, such as by nematodes, triggers their constriction to facilitate prey capture (Schmidt *et al.* 2007). Therefore, it has been proposed that *C. elegans* may elude capture by suppressing their head movements while reversing after they encounter these fungi, allowing them to escape without triggering ring constriction. One of the main neuron pairs involved in the suppression of head movements is the set of tyraminergic RIM interneurons. Specifically, RIM-ablated animals fail to suppress foraging behavior while reversing in response to light anterior touch, suggesting that TA release from the RIMs inhibits these head oscillations (Alkema *et al.* 2005).

Nose touch is primarily sensed by the ASH polymodal nociceptive sensory neurons (with a small contribution from the FLP and OLQ sensory neurons) (Kaplan and Horvitz 1993). The RIM interneurons appear to be part of a “disinhibitory” circuit that serves to tonically dampen locomotor reversals (Piggott *et al.* 2011). In this model, when a relatively weak stimulus (*e.g.*, nose touch) is encountered, the ASH nociceptors signal to the AIBs which, in turn, inhibit the RIMs (Piggott *et al.* 2011). With the RIMs silenced, reversals are enabled through a parallel stimulatory circuit (ASH to the AVA/AVD/AVE command interneurons) (Piggott *et al.* 2011). It was proposed that, in this scenario, the inhibition of RIM also allows for head oscillations (foraging) while animals reverse in response to nose touch (Piggott *et al.* 2011), which would be consistent with the behavioral observations of Alkema *et al.* (2005).

However, it was reported that the RIMs can in fact be stimulated or inhibited by AIB, depending on the strength of the sensory stimulus delivered to the ASH sensory neurons (Piggott *et al.* 2011). For example, the ASHs also detect high osmolarity (Bargmann *et al.* 1990; Kaplan and Horvitz 1993; Hart *et al.* 1999; Hilliard *et al.* 2005), which is a more noxious stimulus than nose touch (Mellem *et al.* 2002). In response to the stronger stimulus of osmotic shock delivered to ASH, AIB stimulates RIM (Piggott *et al.* 2011). The presumed associated release of TA from RIM then inhibits foraging while animals reverse in response to high osmolarity (Piggott *et al.* 2011). We propose that nose touch signaling through ASH can indeed inhibit foraging behavior, as first reported by Rex *et al.* (2004) and repeated here (Figure 4), but that its ability to do so is dependent upon whether the strength of the mechanosensory stimulation is sufficient to elicit tyramine release from RIM. Nuanced differences in the execution of the nose touch assay, including the source and thickness of the hair presented as the obstacle for the “nose-on” collision, influence the efficacy of the nose touch response (D. M. Ferkey, unpublished observations) and likely lead to a difference in signal strength through ASH. We suggest a model in which nose touch is detected by ASH and, if it generates a strong enough signal that AIB promotes tyramine release from RIM, it activates the SER-2 receptors found on *C. elegans* head muscles (Tsalik *et al.* 2003; Rex *et al.* 2004; Donnelly *et al.* 2013; Wilson *et al.* 2017) to inhibit foraging behavior. This model would be consistent with a general need to inhibit foraging while reversing as a survival mechanism, regardless of whether that reversal was triggered by activation of ALM/AVM (anterior touch) or ASH (nose touch or osmotic shock). In each case, activation of the sensory neurons would trigger a reversal response meant to elude danger. Further supporting this model, we note the discrepancy between labs for whether foraging is inhibited while animals reverse in response to high osmolarity; Alkema *et al.* (2005) did not see suppression of foraging in wild-type animals under their assay conditions, while Piggott *et al.* (2011) did. Again, small differences in the set-up or execution of the assay could affect the strength of the signal delivered to the ASHs.

Tyramine receptors have been identified in numerous invertebrate species, including fruit flies (Saudou *et al.* 1990), honeybees (Blenau *et al.* 2000) and cockroaches (Blenau *et al.* 2017), suggesting that tyramine’s role as a neurotransmitter extends beyond *C. elegans*. Like SER-2, each of these receptors also has a putative methylation motif within its third intracellular loop (Figure S1), suggesting that methylation may regulate tyraminergic signaling across multiple phyla. Interestingly, these tyramine receptors, along with the human D2 and *C. elegans* DOP-3 receptors, all signal through Gα_i/o_, which traditionally functions to inhibit adenylyl cyclase and decrease cAMP production (Saudou *et al.* 1990; Malek *et al.* 1993; Voss *et al.* 1993; Vanden Broeck *et al.* 1995; Blenau *et al.* 2000; Bofill-Cardona *et al.* 2000; Rex and Komuniecki 2002; Neve *et al.* 2004; Likhite *et al.* 2015). One possibility is that arginine methylation may preferentially modulate signaling through Gα_i/o_-coupled receptors. However, as only a limited number of receptors have been examined to date, it is also possible that methylation regulates G protein-coupled signaling broadly. Consistent with the latter, many of the GPCRs identified by Likhite *et al.* (2015) to contain a putative methylation motif do not couple with Gα_i/o_.

BLAST (Basic Local Alignment Search Tool) analysis identified the serotonin 1A (5-HT_1A_) receptor as the closest human homolog of the tyraminergic SER-2 receptor (Likhite *et al.* 2015). Similar to the invertebrate TA receptors, the 5-HT_1A_ receptor couples to Gα_i/o_ to mediate inhibitory neurotransmission (Barnes and Sharp 1999). The 5-HT_1A_ receptor also has a putative methylation motif within its third intracellular loop (Figure S1), suggesting that even though the receptor binds a different biogenic amine, its regulation by methylation has likely been conserved through evolution.

Although not examined here, the most recently reported SER-2-regulated behavior is the formation and retrieval of imprinted olfactory memories (Jin *et al.* 2016). Exposing juvenile *C. elegans* to pathogenic bacteria early in their life leads to a long-lasting aversion of the bacteria, with sensory neurons signaling to both AIB and the tyraminergic RIM interneurons to form the imprinted olfactory memory (Jin *et al.* 2016). The RIMs, which are necessary for memory formation, release tyramine that signals through the SER-2 receptor expressed on the AIY interneurons (Jin *et al.* 2016). The SER-2 receptor (and AIY interneurons) is necessary for retrieval of the olfactory memory. However, an additional tyraminergic GPCR, TYRA-2, is also required for imprinted olfactory aversion (Jin *et al.* 2016). We have found that, like SER-2, TYRA-2 contains a putative methylation motif in its third intracellular loop (Figure S1), suggesting that methylation of these receptors may contribute to the formation and/or retrieval of imprinted memories. For example, the introduction of pathogenic bacteria may alter the methylation status of these receptors, perhaps in a cell-specific manner.

The addition of a methyl group to an arginine residue removes a hydrogen bond donor and decreases the electrostatic surface potential at the residue, resulting in a change in size and hydrophobicity that can affect its interaction with binding partners (Bedford and Clarke 2009). Thus, protein arginine methylation plays a key role in regulating protein-protein interactions, and could regulate the activity of GPCRs by modulating the binding (or activation) of G proteins or accessory regulator proteins that interact with the third intracellular loop. Previous studies have also shown that arginine methylation can regulate the local phosphorylation state of target proteins. For example, PRMT1 (a type 1 PRMT) -mediated methylation of FOXO transcription factors (Yamagata *et al.* 2008; Takahashi *et al.* 2011) or BAD (BCL-2 antagonist of cell death) (Sakamaki *et al.* 2011) blocks their phosphorylation by Akt (also known as protein kinase B). In these cases, the methylated arginines lie within the phosphorylation motif. PRMT1-mediated arginine methylation of hnRNPK (heterogeneous nuclear ribonucleoprotein K) also blocks phosphorylation (by PKCδ) of a nearby serine (Yang *et al.* 2014). The predicted arginine methylation motif of the SER-2 receptor (Arg^245^ and Arg^247^) lies between two predicted sites of phosphorylation in the third intracellular loop (Ser^243^ and Thr^250^) (Gattiker *et al.* 2002; Rex *et al.* 2004). Thus, another possibility is that SER-2 methylation by PRMT-5 could regulate the local phosphorylation state of these residues to regulate receptor signaling. Finally, since GPCR phosphorylation can lead to receptor desensitization and subsequent downregulation (Moro *et al.* 1993; Ferguson 2001), methylation of GPCRs could antagonize phosphorylation to regulate cell-surface expression of receptors.

The work described here provides evidence of a third GPCR that is functionally regulated by arginine methylation. In humans, GPCRs are the largest family of tractable drug targets (Overington *et al.* 2006; Lagerström and Schiöth 2008) and are the target of over 30% of all marketed pharmaceuticals (White 2005). Given the therapeutic success associated with targeting enzymes that catalyze the post-translational modification of proteins, such as histone deacetylases (Bose *et al.* 2014), to treat disease, our findings may influence the development of innovative approaches to modulate G protein-coupled signaling for therapeutic benefit. Notably, methylation appears to have a modulatory effect on GPCR signaling, rather than being an absolute requirement for signaling (Likhite *et al.* 2015) (Figures 2-4). Therefore, a new generation of treatments based on manipulating PRMT activity and/or GPCR methylation status (mimicking, promoting or blocking) could allow for finer control over the level of signaling than receptor agonists or antagonists can provide.
